# Behaviour of motor unit action potential rate, estimated from surface EMG, as a measure of muscle activation level

**DOI:** 10.1186/1743-0003-3-15

**Published:** 2006-07-17

**Authors:** Laura AC Kallenberg, Hermie J Hermens

**Affiliations:** 1 Roessingh Research and Development, Enschede, The Netherlands; 2 Faculty of Electrical Engineering, Mathematics and Computer Science, University of Twente, Enschede, The Netherlands

## Abstract

**Background:**

Surface electromyography (EMG) parameters such as root-mean-square value (RMS) are commonly used to assess the muscle activation level that is imposed by the central nervous system (CNS). However, RMS is influenced not only by motor control aspects, but also by peripheral properties of the muscle and recording setup. To assess motor control separately, the number of motor unit action potentials (MUAPs) per second, or MUAP Rate (MR) is a potentially useful measure. MR is the sum of the firing rates of the contributing MUs and as such reflects the two parameters that the CNS uses for motor control: number of MUs and firing rate.

MR can be estimated from multi-channel surface EMG recordings. The objective of this study was to explore the behaviour of estimated MR (eMR) in relation to number of active MUs and firing rate. Furthermore, the influence of parameters related to peripheral muscle properties and recording setup (number of fibers per MU, fiber diameter, thickness of the subcutaneous layer, signal-to-noise-ratio) on eMR was compared with their influence on RMS.

**Methods:**

Physiological parameters were varied in a simulation model that generated multi-channel EMG signals. The behaviour of eMR in simulated conditions was compared with its behaviour in experimental conditions. Experimental data was obtained from the upper trapezius muscle during a shoulder elevation task (20–100 N).

**Results:**

The simulations showed strong, monotonously increasing relations between eMR and number of active MUs and firing rate (r^2 ^> 0.95). Because of unrecognized superimpositions of MUAPs, eMR was substantially lower than the actual MUAP Rate (aMR). The percentage of detected MUAPs decreased with aMR, but the relation between eMR and aMR was rather stable in all simulated conditions. In contrast to RMS, eMR was not affected by number of fibers per MU, fiber diameter and thickness of the subcutaneous layer. Experimental data showed a strong relation between eMR and force (individual second order polynomial regression: 0.96 < r^2 ^< 0.99).

**Conclusion:**

Although the actual number of MUAPs in the signal cannot be accurately extracted with the present method, the stability of the relation between eMR and aMR and its independence of muscle properties make eMR a suitable parameter to assess the input from the CNS to the muscle at low contraction levels non-invasively.

## Background

By means of surface electrodes placed at the skin above a muscle the electrical activity accompanying muscle contractions can be measured non-invasively (surface electromyography, EMG). Parameters based on the amplitude of the signal such as root-mean-square value (RMS) are commonly used in e.g. movement analysis to assess the muscle activation level that is imposed by the central nervous system (CNS) [1-4]. However, RMS is influenced not only by motor control aspects but also by peripheral properties of the muscle such as motor unit (MU) size, as well as by recording setup parameters.

At the single muscle level, motor control is performed by the CNS by regulating the number of active MUs and their firing rate. The number of motor unit action potentials (MUAPs) per second, or MUAP Rate (MR), is the sum of the firing rates of all active MUs and it would therefore directly reflect motor control. In contrast to RMS, MR would not be affected by peripheral muscle fibre properties.

From signals measured with conventional EMG electrodes, arranged in a traditional bipolar configuration, MUAPs can hardly be extracted because of the large number of MUs that contribute to the signal, which consequently results in a high degree of overlap of the MUAPs in the signal. During the past years, several groups have explored the use of array electrodes, consisting of multiple contact points in different configurations (e.g. [5-11].). With such arrays spatial filters can be applied to increase the selectivity of the recording system, thereby decreasing the number of MUs that contribute to the EMG signal. In combination with advanced signal processing techniques, this creates the possibility to examine individual MUAPs in a non-invasive way.

Recently, Gazzoni et al. [12] proposed a method for detection of MUAPs and their classification to the corresponding MUs, that was shown to be able to classify a small but representative sample of MUs. The detection part of this algorithm (based on the Continuous Wavelet Transform; CWT) can be used to obtain an estimate of MR (eMR). A previous study showed significantly higher eMR values in EMG recordings from the upper trapezius during computer tasks in cases with chronic neck-shoulder pain than in healthy controls, while RMS did not show differences [13]. This was attributed to the sensitivity of RMS for peripheral properties and properties of the recording setup, which may have masked differences in motor control.

The objective of this work was to explore to what extent eMR, estimated from the surface EMG by using an electrode array combined with an algorithm based on the CWT, is suitable as a measure of the input of the CNS to a muscle. For this purpose, we investigated 1) the relation between eMR and the two parameters with which the CNS controls muscle activity (number of MUs and firing rate) and 2) to what extent eMR is affected by parameters related to muscle properties and to the recording setup in comparison to RMS.

As information about the actual number of MUAPs in experimental signals is not directly available and physiological variables cannot be controlled experimentally, multi-channel EMG signals were generated with a simulation package. To compare the behaviour of eMR in simulation conditions with its behaviour in experimental conditions, eMR was extracted from experimental multi-channel EMG signals recorded from the upper trapezius muscle during a shoulder elevation task at different force levels.

## Methods

### Simulations

#### Simulation model

To generate EMG signals, a simulation package developed for evaluation of signal processing algorithms for extracting EMG features was used [14]. The model includes the complete transformation from the intracellular action potential to the signal recorded at the surface. First, the extracellular action potential of one muscle fibre is calculated by convoluting an analytical description of the intracellular action potential with a weighting function depending on distance between fibre and detection site, the position along the fibre of the detection site and volume conduction properties. The muscle is modelled as a one-layer cylindrical shape with a high axial and lower radial conductivity. Fat and skin tissue is modelled as a peripheral layer (referred to as subcutaneous layer) where no muscle fibers can be located. Muscle fibers are defined as finite length line sources, located parallel to the skin surface. The muscle fiber conduction velocity is assumed to be linearly related to fiber diameter [15]. Next, a MUAP is obtained by combining the extracellular action potentials of all fibres belonging to one MU. This MUAP is convoluted with a pulse train, resulting in the MUAP train for that MU. Finally, the generated signal consists of the combination of MUAP trains of all contributing MUs. For more details see [14].

Five categories of model parameters can be varied: 1) experimental parameters (describing the detection system), 2) morphological parameters (describing the muscle anatomy), 3) physiological parameters (number of MUs, number of fibres per MU, fibre characteristics), 4) electrical parameters (tissue conductivities) and 5) statistical parameters that define the variability in firing behaviour and in anatomical properties of the MUs.

The algorithm for detection of MUAPs must be applied to a set of signals from adjacent locations in the direction of the muscle fibers, so that propagating MUAPs are identifiable. The configuration of the simulated recording setup was chosen to resemble one row of a two-dimensional electrode array (Helmholtz Institute for Biomedical Engineering, Technical University Aachen, Germany) that was used in the experimental part of the study. It consists of a linear electrode array with 5 contact points (point electrodes) with an inter-electrode distance of 10 mm. The detection area was assumed to be circular. The radius of the detection area (10 mm) was estimated based on [16] and [17]. The simulated location of the electrode array was between the innervation zone and tendon, aligned with the muscle fibre direction.

Morphological, electrical and physiological parameter values were based on data of the biceps brachii (default values of the software package). For a full list of parameter settings, see Table 1.

**Table 1:** Settings of parameters used in the simulation package

Sample frequency	2000 Hz
Signal duration	10 seconds
Muscle length	100 mm
Muscle radius	20 mm
Motor point location	60 mm
Maximal detection distance	10 mm
Electrode diameter	1.8 mm
Intracellular action potential duration	5 ms
Mean muscle fibre conduction velocity	4 m/s
Intracellular conductivity	1.010 S/m
Radial conductivity	0.063 S/m
Longitudinal conductivity	0.330 S/m
Motor unit radius	Mean 4 mm, SD 0.2 mm

#### Simulation protocol

Two sets of simulations were performed: in the first set, the influence of the determinants of MR (number of MUs, firing rate and a combination of both) was investigated while the second set was directed at the influence of parameters, related to peripheral muscle properties and to the recording setup that should affect RMS but not MR (number of fibers per MU, fiber diameter, thickness of the subcutaneous layer, signal to noise ratio).

The simulation protocols are summarised in Table 2. In simulation 1, the number of MUs was varied. To obtain a good estimate of the number of MUAPs in the simulated signals, all MUs had to be located within the detection area of the electrode; else, the number of MUs that contributed to the signal could not be tracked exactly.

**Table 2:** Simulation protocols. Each row represents a simulation. The simulation number is shown in the left column; the settings of all variables are shown in the other columns. In the third simulation, the number of MUs and firing rate are increased simultaneously in steps; each row in the first two columns represents a step.

	**Simulation settings**					
**Simulation number**	**Number of MUs**	**Firing rate (pps)**	**Number of fibers per MU**	**Fibre diameter (μm)**	**Thickness subcutaneous layer (mm)**	**SNR (dB)**

**1**	a: 1–10 in steps of 1, 15–30 in steps of 5 b: 12–120 in steps of 12, 120–300 in steps of 60	Mean: 12, SD: 1	750	55	2	1000
**2**	a: 5 b: 10	Mean: 8 to 20 in steps of 2, SD: 1	750	55	2	1000
**3**	1	10	750	55	2	1000
	2	10				
	4	10				
	5	11				
	6	12				
	7	12.75				
	8	13.5				
	10	14				
	11	15				
	12	16				
**4**	5	Mean: 12, SD: 1	5, 50, 100, 250, 400, 600, 750, 800, 900, 1000	55	2	1000
**5**	5	Mean: 12, SD: 1	750	Mean: 40 to 100 in steps of 10, SD: 5 to 35 in steps of 5	2	1000
**6**	a: 5 b: 10	Mean: 12, SD: 1	750	55	0.5, 1, 2, 3, 4, 5	1000
**7**	a: 5 b: 10 c: 15	Mean: 12, SD: 1	750	55	2	3, 6, 10, 15, 20, 50, 100, 1000

An estimate of the generated number of MUAPs per second in the simulated signal (actual MR, aMR) was estimated by multiplying the number of MUs with the mean firing rate:

*aMR *≈ FR* nrMUs     (1)

Where *FR *= *mean firing rate of all active MUs and nrMUs *= *number of MUs*

**Figure 1:**
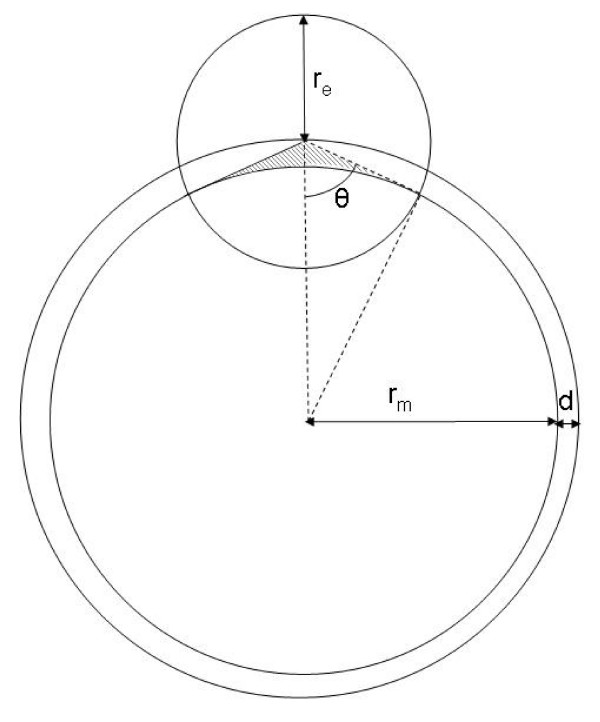
Schematic representation of the muscle and the electrode detection area. Upper circle indicates the electrode detection area, lower circle indicates the muscle and the subcutaneous layer. r_e_: radius of electrode detection area (10 mm), r_m_: muscle radius (20 mm), d: thickness of the subcutaneous layer (2 mm). The ratio between the part of the muscle within the electrode detection area and total muscle cross-section area was calculated for estimation of the number of MUs that contribute to the EMG signal in relation to the total number of MUs, located throughout the muscle. Dotted lines indicate the triangle used for calculation of θ. Shaded area indicates the part of the electrode detection area that lies within the subcutaneous layer and does not contain MUs.

Because the location of the MUs was constrained to the detection area, in the first simulation, the number of MUs was varied over a limited range (from 1 to 30, simulation 1a). To judge the effect of this constraint, in simulation 1b the location of the MUs was *not *restricted to the detection area, and the number of MUs was varied from 12 to 300. In this case, the ratio between the detection area and the muscle cross-section area was included in the estimation of aMR (see Figure 1):

*aMR *≈ FR * nrMUs * RatioAreas     (2)

Where *RatioAreas *= *ratio between part of the muscle within the electrode detection area and total muscle cross-section area*

The detection area contains both skin and muscle tissue. The skin part of the detection area (shaded in Figure 1) is approximately 10%. Because MUs can only be located in the muscle tissue, which is 90% of the detection area, equation (2) becomes:

*aMR *≈ FR * nrMUs *  * 0.9

≈ FR * nrMUs *  * 0.9

Where *r*_*m *_= *muscle radius and r*_*e *_= *detection area radius*

≈ FR * nrMUs *  * 0.9

θ can be calculated with the law of cosine for the triangle indicated in Figure 1 with dotted lines:

*aMR *≈ FR * nrMUs * arccos  * 0.9

Where *d *= *thickness of the subcutaneous layer*

With r_e _= 10 mm, r_m _= 20 mm and d = 2 mm this becomes:

*aMR *≈ FR * nrMUs * 0.069 * 0.9

≈ 0.062 * FR * *nrMUs*

In summary, in simulation 1a, aMR is estimated by FR*nrMUs and in simulation 1b by 0.062*FR*nrMUs.

In simulation 2, firing rate was varied in two conditions: with 5 active MUs (simulation 2a) and with 10 active MUs (simulation 2b). Each MU was assigned an individual firing rate; see Section 2.1.3. Mean firing rate was varied from 8 to 20 pulses per second (pps). In these simulations, all other variables were held constant so that variation in eMR could exclusively be related to variation in one input variable.

In physiological circumstances, the number of MUs and firing rate are not independent of each other. Therefore, in simulation 3 these two variables were varied simultaneously to simulate an increasing force production. Different authors have shown that rate coding mainly contributes to force production at higher force levels (above 30% of the maximal voluntary contraction force, MVC), especially for large muscles [18,19]. Therefore, in the first simulation steps only the number of MUs was increased while in the later steps, both the number of MUs and the mean firing rate were increased simultaneously (see Table 2). The firing rate values were based on experimental research by Conwit et al. [19], who investigated average firing rate in relation to percentage of MVC.

The second set of simulations was directed at the influence of parameters related to peripheral muscle properties and to the recording setup. These parameters do not affect aMR, but they do affect the amplitude and frequency content of the signal. One of the most important peripheral muscle properties is MU size, which is a combination of the number of fibres per MU and their diameter. In simulation 4, the influence of the number of fibers per MU (range 5 – 1000) was investigated while in simulation 5 fiber diameter (40 – 100 μm) was addressed. According to the Henneman principle [20], in physiological circumstances small MUs are always recruited first, and when more force is required, larger MUs are recruited additionally. To simulate this behaviour, the mean and the standard deviation of the distribution from which the mean fibre diameter was drawn were increased simultaneously (see Table 2).

Furthermore, in simulation 6 the influence of thickness of the subcutaneous layer (range 0.1 – 5 mm) was evaluated when 5 MUs (6a) and 10 MUs (6b) were active. Due to filtering effects of the subcutaneous layer, the EMG signal is attenuated [21] and the duration of the MUAPs may become longer, which could lead to an increase in MUAP superimposition. These effects may affect the performance of the algorithm to detect MUAP shapes.

Finally, since the performance of the algorithm was expected to depend on the signal to noise ratio (SNR) as well, this variable was varied from 3 dB to 1000 dB in simulation 7. This simulation was performed with 5, 10 and 15 MUs (simulations 7a – c).

#### Simulation settings

See Table 2. In case the number of MUs was not varied (simulations 2, 4–7), it was set to 5, 10 or 15. The default value of SNR was set to 1000 dB, resembling a signal without noise. The default number of fibres per MU was set to 750, which corresponds to the average MU size in the biceps brachii [22]. Fiber diameter was set to 55 μm and thickness of the subcutaneous layer to 2 mm.

When firing rate was kept constant (simulations 1, 4–7), for each MU, its mean inter-pulse interval (IPI) was drawn from a Gaussian distribution with a mean of 83.3 ms and a standard deviation (SD) of 7 ms (corresponding to a mean firing rate of 12 pps with an SD of 1 pps). The variation within a pulse train (belonging to one MU) was set to ten percent.

The influence of fiber diameter was investigated in simulation 5. For each MU, a mean fibre diameter was drawn from a normal distribution (bounded at ± 3 SDs) with a user-defined mean and SD. Next, the individual fibre diameters within the MU were drawn from a normal distribution (bounded at ± 2 SDs) with the drawn fibre diameter as mean and a SD of 1 μm (default setting of the simulation package).

SNR could not be varied in the simulation package. Therefore, Gaussian noise was added to the simulated signals by using custom-made software written in Matlab (The MathWorks, Inc., Natick, MA, USA).

Each step in the simulations was repeated three times and outcome values were averaged to decrease the variability introduced in the input parameters.

### Experimental set-up

#### Subjects

The study was approved by the local medical ethics committee. Five subjects (three female, two male, mean (SD) age 26.6 (2.70) years, weight 68.4 (10.9) kg, height 175.8 (11.3) cm, body-mass index (BMI) 22.1 (1.9) kg/m^2^) without known disorders took part in this study. All subjects gave their written informed consent.

#### General procedures

Subjects performed a stepwise increasing contraction consisting of five force levels of 20 to 100 N in steps of 20 N. The force levels were shown on a laptop screen and subjects were instructed to keep the force level as constant as possible for each step. Each level was maintained for ten seconds. Between the levels, one second was allowed for transition to the next level.

Subjects were seated on a chair that was adjusted in height to prevent them from touching the floor with their feet. The chair was attached to a frame that was fixed to the wall. Two force transducers (Thermonobel, Karlskoga, Sweden) were attached to the frame for measuring the force from the trapezius muscle. The position of the force sensors was adjusted to body size, such that the sensor centre was located slightly above the acromion. In rest, the force sensors were just not touching the subject. The force signals were sampled with 1 kHz and digitised with a 16-bits A/D converter, and stored on a laptop.

Subjects were instructed not to speak or move the head during the recordings, to sit straight, and to keep their hands rested in the lap. Subjects were not allowed to cross their feet.

#### EMG recordings

EMG of the dominant upper trapezius was recorded using a two-dimensional 16-channel electrode array (Helmholtz Institute for Biomedical Engineering, Technical University Aachen, Aachen, Germany). The array consisted of four rows, the first and fourth containing three contact points and the middle two containing five contact points. The distance between the rows was 10 mm, as was the distance between the adjacent electrodes within a row. The inter-electrode distance is relatively small in comparison with conventional surface EMG measurements, which increases the spatial selectivity.

Before electrode placement, the skin was cleaned using abrasive paste. Electrodes were placed with the rows parallel to the line from the spinous process of the seventh cervical vertebra (C7) to the acromion with the centre of the electrode 2 cm lateral from the midpoint, in accordance with the SENIAM recommendations [23]. A ground electrode was placed on the wrist of the dominant side. The monopolar signals were amplified 1000 times, sampled at 4000 Hz and band-pass filtered (10–500 Hz) with a custom made EMG amplifier (Helmholtz Institute for Biomedical Engineering, Technical University Aachen, Aachen, Germany). The signals were digitised using a 16 bit A/D-converter and stored on a laptop. Before the measurement started, the signal quality was inspected visually. Criteria for correct electrode placement were presence of propagating MUAPs across the channels, similarity of the MUAP shapes in all channels and absence of excessive noise. Adjustments were made when necessary until signals with good quality could be obtained.

### Data analysis

Monopolar signals with an inter-electrode distance of 10 mm from adjacent electrodes from the middle two rows of the array were subtracted, resulting in two sets of four single differential signals. For both sets, cross-correlation between adjacent signals was calculated, resulting in three values from each set. Adjacent signals are expected to show a high degree of similarity when there are no artefacts present. The set with the highest average correlation coefficient was therefore selected for further processing.

For the simulated signals, analogous to the experimental signals, a set of four single differential signals was constructed by subtracting signals from adjacent electrodes.

For detection of MUAPs, a wavelet-based algorithm that uses multi-channel information was applied ([12,24]). The algorithm uses the continuous wavelet transform (CWT) to identify shapes that are similar to a mother wavelet. As mother wavelet, the first Hermite-Rodriguez function was used. The CWT uses two parameters, being a time shift (related to the location in time where a similar shape occurred) and a scale factor that is related to the amplitude and width of the wavelet. The CWT of each single signal is calculated for a range of different values for both parameters. The squared output of the CWT (ranging from 0 to 1) is a measure for the similarity between the mother wavelet and the signal at a certain time instant. This output can be plotted in a three-dimensional graph against the time instant and the scale factor, resulting in a so-called scalogram.

The algorithm started with calculating the CWT for the first channel. When the scalogram reached a maximum that was higher than a user-defined threshold (set to 0.1 in this study), a candidate MUAP was found at the time instant and scale factor corresponding to the maximum. The algorithm then searched for candidate MUAPs that were located in the surrounding channels within a time delay corresponding to a conduction velocity between 2 and 8 m/s. When the candidate was present in a minimal number of channels (set to 3 in this study), the candidate was considered a MUAP. Then, the CWT was calculated for the next channel. The algorithm cycled through the channels in this way. Outputs of the algorithm were the firing times and the corresponding MUAP shapes on each channel. For more details, see [12,24].

From the firing instances, the number of MUAPs (resulting from all MUs together) was extracted for time windows of one second. The mean value (across time) was calculated and is reported as eMR. aMR is estimated by multiplying the average firing rate with the number of MUs.

Root-mean-square values (RMS) were calculated from each signal for time windows of one second. Values were calculated for each channel and averaged both across channels and across time.

The algorithms were implemented in Matlab software (The MathWorks, Inc., Natick, MA, USA).

## Results

Throughout the results section, eMR, aMR and RMS are compared.

**Figure 2:**
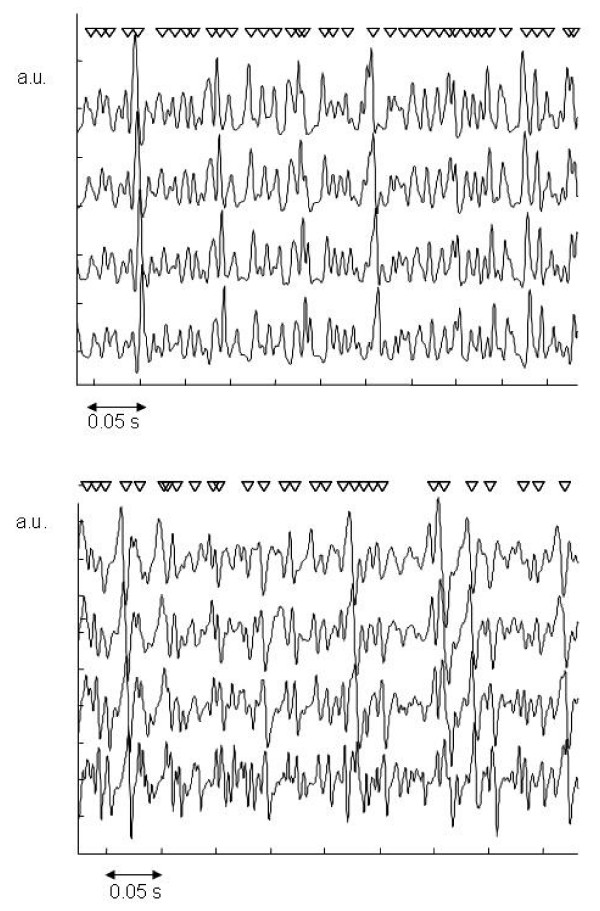
Simulated signals with ten active MUs (upper graph) and experimentally recorded signal at a force level of 100 N (lower graph). Four single differential signals with 10 mm inter-electrode distance, recorded parallel to the muscle fibers are shown. Fibre direction is from innervation zone (upper signal) to tendon (lower signal). Triangles indicate detected MUAPs. A.u.: arbitrary units.

In Figure 2, an example of a simulated signal is shown for 10 active MUs, together with an example of an experimentally recorded signal from the upper trapezius muscle at 100 N for comparison. The appearance of the simulated signal is similar to the experimentally recorded signal. The median frequency of the power spectrum of the simulated signals (first channel) is 64.7 Hz, while that of the experimental signal (first channel) is 63.5 Hz. When less active MUs are simulated, individual MUAPs can easily be recognised.

### Determinants of MR

**Figure 3:**
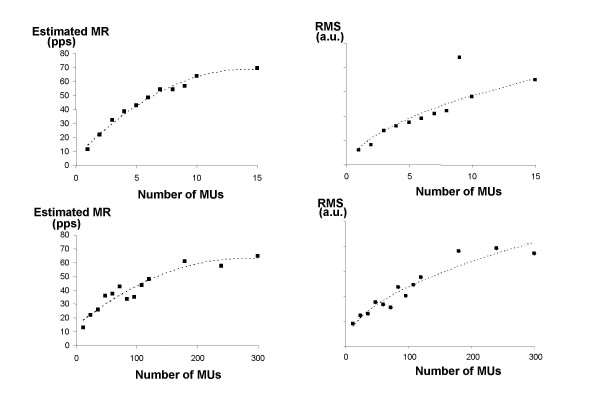
Relation between number of active MUs and both estimated MR and RMS in simulated conditions. Upper graphs show the relations when MUs were restricted to be located within detection area of electrode. Lower graphs show the relations when MUs were located throughout the whole muscle. Scales of the y-axis are the same in both RMS graphs.

In Figure 3 (upper graphs), the relation between the number of active MUs and both eMR and RMS when the MUs are located within the detection area of the electrodes is shown (simulation 1a). eMR increases with the number of active MUs, but the percentage of detected MUAPs decreases. Visual inspection of the signals underlines that this is related to the increasing occurrence of superimpositions that are detected as single MUAPs. The best fit of a second order polynomial trend line resulted in an explained variance (squared Pearson's correlation coefficient, r^2^) of 0.99 (p < 0.001).

RMS also increases with number of active MUs. The best trend line was a square root relation which resulted in an explained variance of 0.86 (p < 0.001).

Figure 3 also shows the relation between number of MUs and both eMR and RMS when the location of the MUs was not restricted to the detection area (simulation 1b, lower graphs). The shape of the curve is similar as for simulation 1a, but the variability of the measurements is larger, as is reflected in the somewhat lower explained variance: the best fit was a second order polynomial trend line with an explained variance of 0.91 (p < 0.001). RMS was best approximated by a square root relation, with an explained variance of 0.92 (p < 0.001).

In simulation 2 firing rate was simulated in two conditions: 1) while 5 MUs are active, 2) while 10 MUs are active. aMR increases linearly with firing rate in both situations, with a steeper slope when 10 MUs are active. eMR increases linearly as well, but the slope of the curve is less steep than for aMR. Fitting of a linear regression line through the eMR curves resulted in a line with a slope of 2.18 and an intercept of 41.7 pps (r^2 ^= 0.96, p < 0.001) for 5 active MUs and a slope of 1.72 and an intercept of 16.6 pps (r^2 ^= 0.95, p < 0.0001) for 10 active MUs. The curve for 10 active MUs is shifted to higher values than the curve for 5 active MUs.

**Figure 4:**
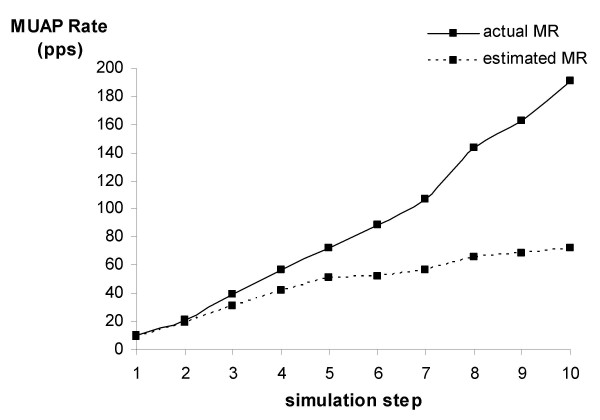
Actual and estimated MR in relation to simulated force production. To simulate an increasing force, the number of MUs and their firing rate were increased simultaneously. See Table 2 for the parameter values at each step.

Figure 4 shows the behaviour of MR when increasing force production is simulated as a combined increase of firing rate and number of MUs. Both aMR and eMR increase with simulated force. The increase is less for eMR than for aMR, similar to the results of simulation 1 and 2.

**Figure 5:**
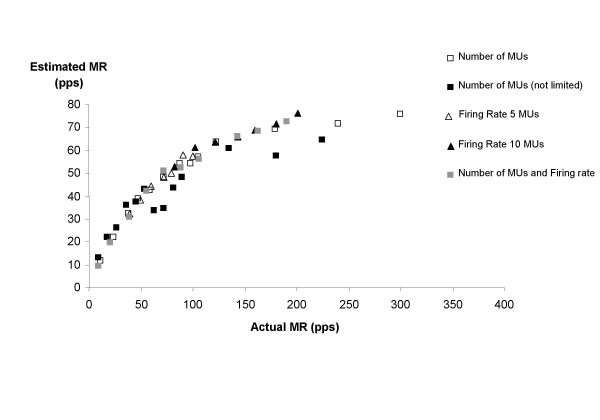
Relation between actual and estimated MR in different conditions. Results of simulations with varying number of active MUs, firing rate, and a combination of both. The relations with number of MUs were simulated in two conditions: when MUs were restricted to be located within the detection area of the electrode and when MUs were located throughout the whole muscle (indicated as "number of MUs (not limited)" in the legend). The relations with firing rate were investigated in case of 5 and 10 active MUs.

In Figure 5, the influence of the determinants (number of MUs and firing rate) on eMR in different conditions is summarized. This figure provides an impression of the stability of the relation between eMR and aMR in different conditions. It shows that this relation is very similar for the different simulations. The results from simulation 1b (number of MUs with MUs distributed across the whole muscle) deviate somewhat from the curve with slightly lower eMR values, but the shape of the relation is similar. For the pooled data, a logarithmic trend line resulted in an explained variance of 0.94 while a second order polynomial trend line resulted in r^2 ^= 0.92.

### Parameters related to muscle properties and recording setup

Except from the relation between RMS and thickness of the subcutaneous layer, the relations between aMR, eMR and RMS on one hand and number of fibers, fiber diameter and thickness of the subcutaneous layer on the other hand were best approximated with a linear fit. Linear regression analysis was applied to estimate the coefficients of the relations, and the explained variance. In contrast, the relation between RMS and thickness of the subcutaneous layer was obviously non-linear. This relation could best be approximated by a logarithmic relation. Explained variance and coefficients were in this case estimated with non-linear regression. Table 3 shows that number of fibers, fiber diameter and thickness of the subcutaneous layer explain a high percentage of variance of RMS values (r^2 ^> 0.94) but not of eMR and aMR (r^2 ^< 0.13). There is no significant in- or decrease in aMR and eMR with these parameters, while RMS increases strongly with number of fibers and fiber diameter. RMS decreases logarithmically with thickness of the subcutaneous layer.

**Table 3:** Influence of peripheral properties on aMR, eMR and RMS. Linear regression was applied for estimation of the percentage of explained variance (r^2^) and of the intercept β0 and slope β1. The relation between RMS and thickness of the subcutaneous layer could best be approximated with a logarithmic relation. Nonlinear regression was performed to estimate the coefficients of this relation.

		aMR (pps)				eMR (pps)				RMS (a.u.)			
		
		β0	β1	r^2^	p	β0	β1	r^2^	p	β0	β1	r^2^	p
Number of fibers		62.2	-0.0011	0.11	0.35	41.4	0.0027	0.13	0.31	1.15	0.14	0.96	0.001
Fiber diameter		57.2	0.018	0.046	0.65	37.0	0.048	0.11	0.47	118	4.5	0.97	0.001
Thickness of subcutaneous layer	5 MUs	59.9	0.072	0.038	0.57	42.5	0.24	0.062	0.46	132	-37	0.94	0.001
Thickness of subcutaneous layer	10 MUs	122	-0.36	0.073	0.42	61.3	0.31	0.027	0.63	195	-63	0.98	0.001

The aMR and corresponding eMR intercept values (β0) that were found in simulations 4 tot 7 are consistent with the relation between eMR and aMR as was found in simulations 1 to 3 (Figure 5).

**Figure 6:**
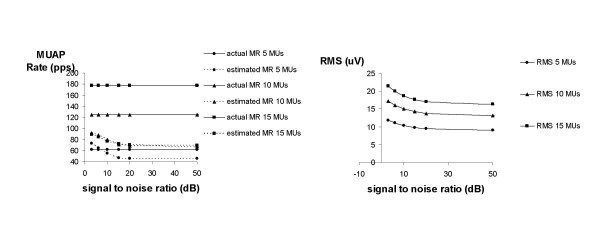
Influence of signal to noise ratio on estimated MR. Simulations were performed in case of 5, 10 and 15 active MUs.

The influence of signal-to-noise ratio is shown in Figure 6 for 5, 10 and 15 active MUs. Obviously, aMR does not change with SNR. For values lower than 15 dB, eMR increases. In case of 5 active MUs, eMR is even higher than aMR. RMS shows a similar behaviour.

### Experimental results

**Figure 7:**
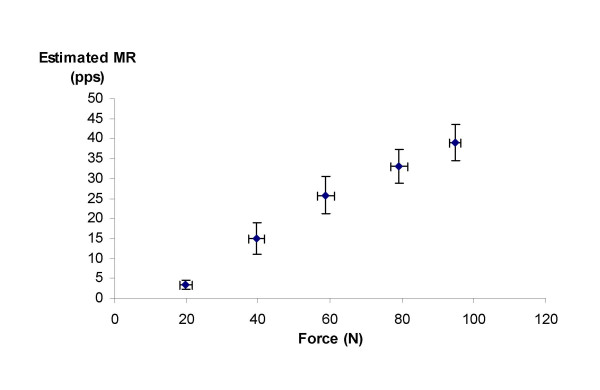
Relation between estimated MR and force in experimental conditions during a step contraction of the trapezius muscle (force levels from 20 to 100 N). Mean values of 5 subjects are shown. Bars show inter-subject standard errors of the mean both in force and estimated MR.

The experimental results are reported in Figure 7. The relation between eMR and force is approximately linear, although the increase in eMR flattens for the force levels of 80 and 100 N. Individual second order polynomial trend lines resulted in an average explained variance of 0.98 (range 0.97–0.99, p < 0.001). Linear trend lines explained slightly less variance (mean r^2 ^= 0.94, range 0.88–0.97).

## Discussion

The objective of this work was to explore to what extent eMR, estimated from the surface EMG by using an electrode array combined with an algorithm based on the continuous wavelet transform, is suitable as a measure of the input of the CNS to a muscle. For this purpose, we investigated 1) the relation between eMR and the two parameters with which the CNS controls muscle activity (number of MUs and firing rate) and 2) the influence of parameters related to muscle properties and to the recording setup on eMR in comparison to RMS.

### Determinants of MR

In simulations 1 to 3, the influence of the number of MUs and firing rate on eMR were investigated. The high percentages of explained variance show that although eMR diverges widely from aMR, eMR is strongly related to number of active MUs (simulation 1) and firing rate (simulation 2), as well as to a combination of both (simulation 3). The results from the different simulations are consistent (Figure 5), which gives an indication of the stability of the relation between aMR and eMR. Increases in the number of MUs and firing rate seem to be interchangeable; eMR only depends on the total number of MUAPs per second.

The increase of eMR with number of MUs could well be approximated (r^2 ^= 0.99) by a second order polynomial fit with a negative coefficient for the quadratic term. This indicates that the percentage of detected MUAPs decreases when the number of MUs increases. Visual inspection of the signals reveals that this is related to the occurrence of superimpositions that are either not recognized, or detected as single MUAPs. Assuming that the number of superimpositions increases linearly, the percentage of detected MUAPs decreases linearly as well, which would indeed result in a second order polynomial relation. Several algorithms aiming at full EMG decomposition contain a method for resolving superimpositions [25-28] These algorithms are developed for invasive needle or wire recordings and are based on the shape differences between MUAPs from different MUs. However, for surface EMG recordings, the MUAP shapes from different MUs are rather similar. Other approaches to resolve superimpositions such as algorithms based on independent component analysis [29,30], that do not necessarily rely on the occurrence of temporally isolated MUAPs in the signal may prove to be more successful.

In order to make a reliable estimate of aMR, MUs were restricted to be located within the detection area. When the location of the MUs was not restricted, the variability of both RMS and eMR was higher. Probably, part of this variability is related to errors in the estimate of the number of MUs that contribute to the signal. MUs may partly lie within the detection area and it depends on the location of the center of the MU whether it is included in the estimate of the number of MUs or not. Furthermore, contribution of parts of MUs is likely to increase background activity. However, despite the increased variability, the shape of the relation between eMR and number of MUs was the same for simulations 1a and 1b. Thus, the restriction of the location of MUs to the detection area of the electrode had a rather limited effect.

In conclusion, the simulation results show that eMR considerably diverges from aMR. This implies that eMR cannot directly be used to estimate the true number of MUAPs in the EMG signal. However, the relation between eMR and aMR is rather stable in different conditions and eMR is strongly related to the number of MUs and firing rate.

### Parameters related to muscle properties and recording setup

In contrast to RMS, eMR was not affected by number of fibers per MU, fiber diameter and thickness of the subcutaneous layer. This underlines that eMR specifically reflects parameters related to the input of the CNS to the muscle, whereas RMS also depends on peripheral muscle and subcutaneous layer properties. In a previous study differences between cases with chronic neck-shoulder pain and healthy controls were found in eMR, while RMS did not show any differences [13]. The sensitivity of RMS for peripheral properties, that the present findings confirm, was suggested to be a cause of this. The influence of peripheral properties may have masked subtle differences in motor control. The sensitivity of RMS for peripheral properties may be decreased by normalising the RMS values to an individual's RMS value during MVC, as is often done in experimental studies. However, especially in subjects with pain or fear of pain, it may be difficult to assess an individual's maximal capacity reliably.

Two aspects of MU size were investigated: the number of fibers per MU and fiber diameter. Both parameters did not affect eMR, while they showed a linear relation with RMS. Because in physiological circumstances, additionally recruited MUs in general will be larger [20], MU size also affects the relation between RMS and force. The sensitivity of RMS for peripheral properties in general may lead to a higher inter-subject variability for RMS than for eMR.

The percentage of detected MUAPs remained constant when fiber diameter was varied. By increasing the mean muscle fibre diameter within a MU as well as the width of the distribution of mean fibre diameter across MUs simultaneously, the recruitment of larger MUs was simulated while the smaller MUs remained present in the signal. In this way it was shown that small MUAPs are still detected in the presence of large MUAPs. This is in agreement with simulation results of Gazzoni et al. [12], who also reported that both small and large MUs were simultaneously detected.

It was shown that estimation of MR is hampered when the SNR becomes lower than 15 dB, independent of the number of active MUs. In this case, apparently noise is generating false positives. RMS was also over-estimated for lower SNRs. This implies that the SNR in experimental conditions should be higher than 15 dB. In the experimental part of this study SNR (estimated from the signal variance during contraction divided by the signal variance during rest) typically ranged from 40 to 100 dB for the applied force range, indicating that noise did not hamper the estimation of MR.

### Experimental results

The experimental results showed strong, second order polynomial individual relations between eMR and contraction force (0.97 < r^2 ^< 0.99). In comparison, individual linear relations between RMS and shoulder elevation torque with explained variances of 88–97% have been reported [31].

The maximal force that was measured was 100 N, which corresponded to an eMR of approximately 40 pps. From Figure 5 can be seen that in the range from 0 to 40 pps, the increase of eMR is approximately linear. A force of 100 N corresponds to 25–30% of MVC, that was 357 N for healthy subjects in the same experimental setup [32]. For higher force levels, the eMR-force curve will probably flatten, due to the increased occurrence of superimpositions.

Absolute rather than relative force levels were used in this study, since in daily life conditions, experienced loads are also not scaled to an individual's capacity. Relative force levels are often used to decrease inter-subject variability. When the force levels would have been normalised, the relation between eMR and force might have been even stronger.

The number of MUs that contribute to the signal is strongly dependent on the spatial selectivity of the recording system [5]. Selection of the recording system involves a trade-off between representation of all MUs and optimal MR estimation. The single differential configuration with the relatively small inter-electrode distance (10 mm) that was applied for this study appeared to be suitable for the range of investigated force levels. For higher force levels, MR estimation might improve by applying a more spatially selective filter, which can be reached with a more selective electrode configuration or with a smaller inter-electrode distance. When recordings are made with a two-dimensional array, as was done in this study, the spatial selectivity can be increased by using the Laplacian configuration [7]. With linear electrode arrays [8], the inter-electrode distance could be shortened.

### Methodological aspects

MR is a combination of the number of active MUs and their firing rates and does not give information about each of these variables separately. Many research groups are working on algorithms for complete EMG decomposition (e.g. [12,33-35]). In most algorithms, the first step of decomposition is the detection of MUAPs in the signal. The second step consists of the assignment of the detected MUAPs to the MU that generated them (classification). Other algorithms are based on higher-order statistical features of the EMG signals [29].

Complete decomposition would result in clinically relevant information that can easily be interpreted. However, most methods are only able to decompose very few MUs (about 5) completely from surface EMG signals. Furthermore, decomposition of MUs with small MUAP amplitude is difficult, whereas the results of simulation 5 show that detection of small MUAPs is possible even in the presence of big MUAPs.

Zhou et al. also developed a method for MUAP counting based on template matching [10]. They obtained MUAP templates of each MU from spike-triggered averaging of the surface EMG signal by using decomposed intramuscular EMG signals as trigger. These templates were then used to generate a simulated EMG signal. Their MUAP counting algorithm was able to estimate MR reliably up to 100 pps from these signals, which seems a better performance than that of the algorithm we applied. For higher values, the performance of the algorithm also decreased. It should be taken into account that the templates used for generation of simulated signals were used for detection as well in the algorithm of Zhou et al. Since such templates are not a priori known in an experimental setting without intramuscular recordings, the performance of the algorithm in such conditions remains to be investigated.

## Conclusion

The simulations showed rather stable, monotonously increasing relations between eMR and both number of active MUs and firing rate. In contrast to RMS, eMR is hardly influenced by the number of fibers per MU, fiber diameter and thickness of the subcutaneous layer. eMR therefore seems to specifically reflect input from the central nervous system to the muscle while it is not affected by peripheral aspects. Experimental data showed a strong, approximately linear relation between eMR and force.

Although the actual number of MUAPs in the signal cannot be accurately extracted with the present method, eMR seems to be a suitable non-invasive tool to study the input of the central nervous system to the muscle at low contraction levels.

## Competing interests

The author(s) declare that they have no competing interests.

## Authors' contributions

LK participated in the conception and design of the study, carried out the experimental part of the study, analysed and interpreted the data and drafted the manuscript. HH participated in the conception and design of the study, helped in interpreting the data and revised the manuscript. All authors read and approved the final manuscript.

## References

[B1] Winter DA, Yack HJ (1987). EMG profiles during normal human walking: stride-to-stride and inter-subject variability. Electroencephalogr Clin Neurophysiol.

[B2] Mathiassen SE, Aminoff T (1997). Motor control and cardiovascular responses during isoelectric contractions of the upper trapezius muscle: evidence for individual adaptation strategies. Eur J Appl Physiol Occup Physiol.

[B3] Bilodeau M, Schindler-Ivens S, Williams DM, Chandran R, Sharma SS (2003). EMG frequency content changes with increasing force and during fatigue in the quadriceps femoris muscle of men and women. J Electromyogr Kinesiol.

[B4] Farina D, Madeleine P, Graven-Nielsen T, Merletti R, Arendt-Nielsen L (2002). Standardising surface electromyogram recordings for assessment of activity and fatigue in the human upper trapezius muscle. Eur J Appl Physiol.

[B5] Disselhorst-Klug C, Silny J, Rau G (1997). Improvement of spatial resolution in surface-EMG: a theoretical and experimental comparison of different spatial filters. IEEE Trans Biomed Eng.

[B6] Zwarts MJ, Stegeman DF (2003). Multichannel surface EMG: basic aspects and clinical utility. Muscle Nerve.

[B7] Farina D, Schulte E, Merletti R, Rau G, Disselhorst-Klug C (2003). Single motor unit analysis from spatially filtered surface electromyogram signals. Part I: spatial selectivity. Med Biol Eng Comput.

[B8] Merletti R, Farina D, Gazzoni M (2003). The linear electrode array: a useful tool with many applications. J Electromyogr Kinesiol.

[B9] Blok JH, van Dijk JP, Drost G, Zwarts MJ, Stegeman DF (2002). A high-density multichannel surface electromyography system for the characterization of single motor units. Rev Sci Instrum.

[B10] Zhou P, Rymer WZ (2004). MUAP number estimates in surface EMG: template-matching methods and their performance boundaries. Ann Biomed Eng.

[B11] Roeleveld K, Stegeman DF, Vingerhoets HM, Van Oosterom A (1997). The motor unit potential distribution over the skin surface and its use in estimating the motor unit location. Acta Physiol Scand.

[B12] Gazzoni M, Farina D, Merletti R (2004). A new method for the extraction and classification of single motor unit action potentials from surface EMG signals. J Neurosci Methods.

[B13] Kallenberg LA, Hermens HJ (2006). Motor unit action potential rate and motor unit action potential shape properties in subjects with work-related chronic pain. Eur J Appl Physiol.

[B14] Duchene J, Hogrel JY (2000). A model of EMG generation. IEEE Trans Biomed Eng.

[B15] Nandedkar SD, Stalberg E (1983). Simulation of single muscle fibre action potentials. Med Biol Eng Comput.

[B16] Roeleveld K, Stegeman DF, Vingerhoets HM, Van Oosterom A (1997). Motor unit potential contribution to surface electromyography. Acta Physiol Scand.

[B17] Barkhaus PE, Nandedkar SD (1994). Recording characteristics of the surface EMG electrodes. Muscle Nerve.

[B18] Kukulka CG, Clamann HP (1981). Comparison of the recruitment and discharge properties of motor units in human brachial biceps and adductor pollicis during isometric contractions. Brain Res.

[B19] Conwit RA, Stashuk D, Tracy B, McHugh M, Brown WF, Metter EJ (1999). The relationship of motor unit size, firing rate and force. Clin Neurophysiol.

[B20] Henneman E, Somjen G, Carpenter DO (1965). Functional significance of cell size in spinal motoneurons. J Neurophysiol.

[B21] Nordander C, Willner J, Hansson GA, Larsson B, Unge J, Granquist L, Skerfving S (2003). Influence of the subcutaneous fat layer, as measured by ultrasound, skinfold calipers and BMI, on the EMG amplitude. Eur J Appl Physiol.

[B22] Buchthal F (1961). The general concept of the motor unit. Neuromuscular disorders. Res Publ Assoc Res Nerv Ment Dis.

[B23] Hermens HJ, Freriks B, Disselhorst-Klug C, Rau G (2000). Development of recommendations for SEMG sensors and sensor placement procedures. J Electromyogr Kinesiol.

[B24] Farina D, Fortunato E, Merletti R (2000). Noninvasive estimation of motor unit conduction velocity distribution using linear electrode arrays. IEEE Trans Biomed Eng.

[B25] Stashuk D (2001). EMG signal decomposition: how can it be accomplished and used?. J Electromyogr Kinesiol.

[B26] McGill KC, Lateva ZC, Marateb HR (2005). EMGLAB: An interactive EMG decomposition program. J Neurosci Methods.

[B27] Loudon GH, Jones NB, Sehmi AS (1992). New signal processing techniques for the decomposition of EMG signals. Med Biol Eng Comput.

[B28] Zennaro D, Wellig P, Koch VM, Moschytz GS, Laubli T (2003). A software package for the decomposition of long-term multichannel EMG signals using wavelet coefficients. IEEE Trans Biomed Eng.

[B29] Nakamura H, Yoshida M, Kotani M, Akazawa K, Moritani T (2004). The application of independent component analysis to the multi-channel surface electromyographic signals for separation of motor unit action potential trains: part I-measuring techniques. J Electromyogr Kinesiol.

[B30] Nakamura H, Yoshida M, Kotani M, Akazawa K, Moritani T (2004). The application of independent component analysis to the multi-channel surface electromyographic signals for separation of motor unit action potential trains: part II-modelling interpretation. J Electromyogr Kinesiol.

[B31] Louhevaara V, Long A, Owen P, Aickin C, McPhee B (1990). Local muscle and circulatory strain in load lifting, carrying and holding tasks. Int J Ind Ergon.

[B32] Schulte E, Kallenberg LA, Christensen H, Disselhorst-Klug C, Hermens HJ, Rau G, Sogaard K (2006). Comparison of the electromyographic activity in the upper trapezius and biceps brachii muscle in subjects with muscular disorders: a pilot study. Eur J Appl Physiol.

[B33] Kleine BU, Blok JH, Oostenveld R, Praamstra P, Stegeman DF (2000). Magnetic stimulation-induced modulations of motor unit firings extracted from multi-channel surface EMG. Muscle Nerve.

[B34] Chauvet E, Fokapu O, Hogrel JY, Gamet D, Duchene J (2003). Automatic identification of motor unit action potential trains from electromyographic signals using fuzzy techniques. Med Biol Eng Comput.

[B35] Holobar A, Zazula D (2004). Correlation-based decomposition of surface electromyograms at low contraction forces. Med Biol Eng Comput.

